# Supplementary material for: Causal Relationship between Aging and Anorexia Nervosa: A White-Matter-Microstructure-Mediated Mendelian Randomization Analysis
Source: Biomedicines. 2024 Aug 16;12(8):1874. doi: 10.3390/biomedicines12081874 (PMC11351342; doi:10.3390/biomedicines12081874)
Supplement: Supplementary file 1 [file biomedicines-12-01874-s001.zip › Supplementary Figure/Figure S9/Leaveoneout plot of MR analysis for ubm-b-1875 to AN.pdf]

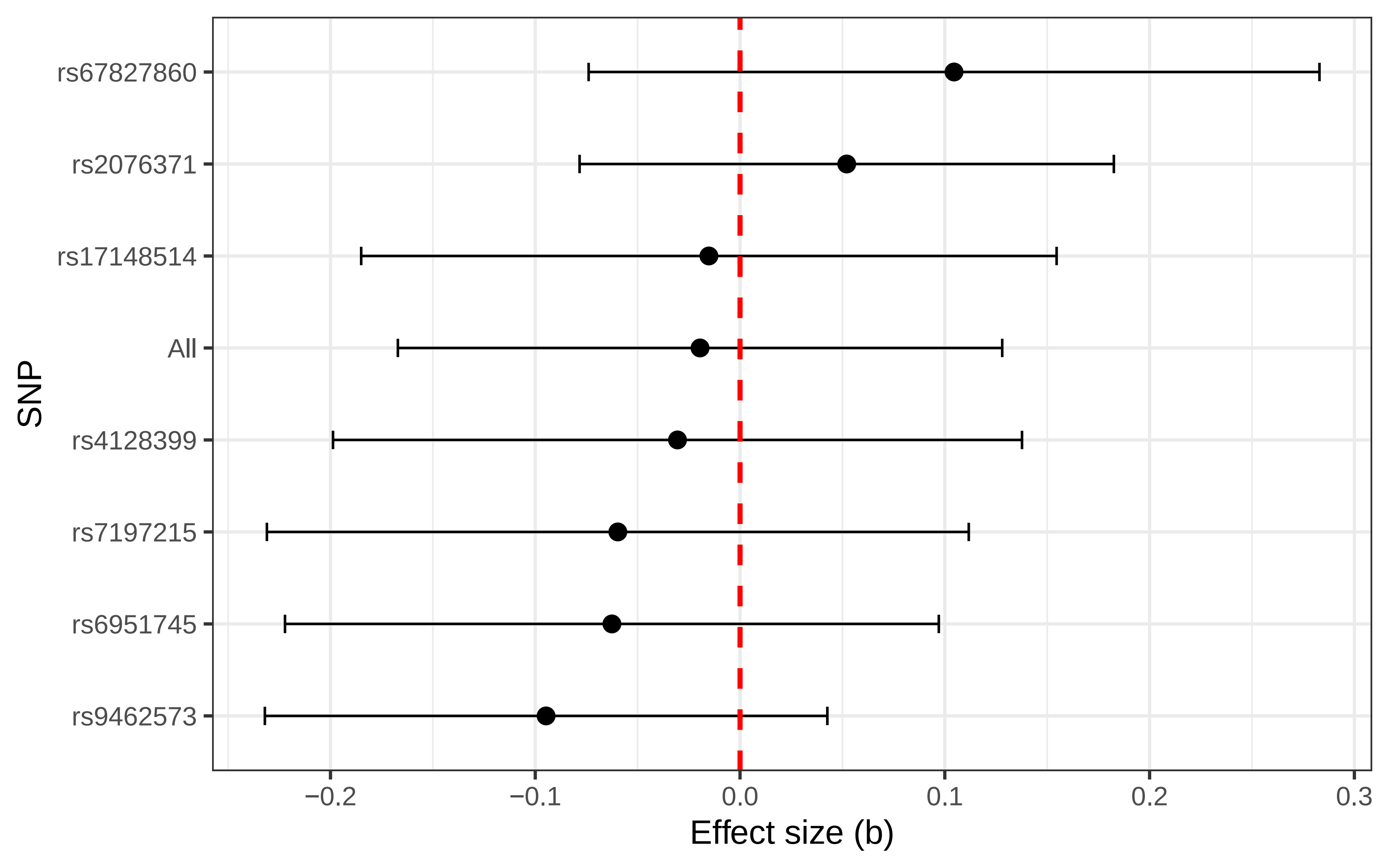

rs67827860
rs2076371
rs17148514
All
rs4128399
rs7197215
rs6951745
rs9462573
SNP
−0.2
−0.1
0.0
0.1
0.2
0.3
Effect size (b)
